# The linear symmetries of Hill’s lunar problem

**DOI:** 10.1007/s00013-022-01822-1

**Published:** 2023-01-16

**Authors:** Cengiz Aydin

**Affiliations:** Institut de mathématiques, Université de Neuchâtel, Rue Emile-Argand 11, 2000 Neuchâtel, Switzerland

**Keywords:** Symmetry, Three body problem, Hill lunar problem, 37J06, 70H33

## Abstract

A symmetry of a Hamiltonian system is a symplectic or anti-symplectic involution which leaves the Hamiltonian invariant. For the planar and spatial Hill lunar problem, four resp. eight linear symmetries are well-known. Algebraically, the planar ones form a Klein four-group ${\mathbb {Z}}_2 \times {\mathbb {Z}}_2$ and the spatial ones form the group ${\mathbb {Z}}_2 \times {\mathbb {Z}}_2 \times {\mathbb {Z}}_2$. We prove that there are no other linear symmetries. Remarkably, in Hill’s system the spatial linear symmetries determine already the planar linear symmetries.

## Introduction and result

**Fig. 1:**
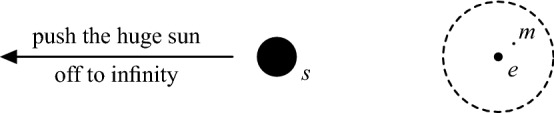
Hill’s lunar problem

Hill’s lunar problem is a limit case of the restricted three-body problem. In the restricted three-body problem, one considers two massive primaries and a massless object which does not influence the two masses and is attracted by them according to Newton’s law of gravitation. In Hill’s original set-up from 1877 [7], the primaries are the sun and the earth, and the massless body is the moon. The goal is to understand the dynamics of the moon. In Hill’s system, the mass of the sun is infinitely much heavier than the mass of the earth, while the moon gets very close to the earth. Therefore, one shifts the earth to the origin and zooms in a region around the earth by pushing the huge sun off to infinity (see Figure 1).

There are two versions of Hill’s lunar problem, the planar and the spatial one. The Hamiltonian describing the motion of the moon in the planar problem is

1 $$\begin{aligned}{} & {} H_p :T^* \big ( {\mathbb {R}}^2 \setminus \{ (0,0) \} \big ) \rightarrow {\mathbb {R}},\nonumber \\{} & {} \quad (q,p) \mapsto \frac{1}{2}|p|^2 - \frac{1}{|q|} + p_1q_2 - p_2q_1 - q_1^2 + \frac{1}{2}q_2^2, \end{aligned}$$

and in the spatial problem,

2 $$\begin{aligned}{} & {} H :T^* \big ( {\mathbb {R}}^3 \setminus \{ (0,0,0) \} \big ) \rightarrow {\mathbb {R}},\nonumber \\{} & {} \quad (q,p) \mapsto \frac{1}{2}|p|^2 - \frac{1}{|q|} + p_1q_2 - p_2q_1 - q_1^2 + \frac{1}{2}q_2^2 + \frac{1}{2}q_3^2, \end{aligned}$$

where each phase space is endowed with the canonical symplectic form $\omega = \sum dq_i \wedge dp_i$. For the derivation of the Hamiltonians, we refer to [4, pp. 77–78] for the planar problem, and to [2, pp. 3–4] for the spatial problem.

A symmetry $\rho$ is, by definition, a symplectic or anti-symplectic involution of the phase space which leaves the Hamiltonian invariant, i.e.,

$$\begin{aligned} H \circ \rho = H,\quad \rho ^2 = \text {id},\quad \rho ^* \omega = \pm \omega . \end{aligned}$$

Symmetries of Hill’s lunar problem play an important role since a natural class of periodic orbits are those that are invariant with respect to such involutions. In fact, linear symmetries are used traditionally for finding and studying invariant orbits; analytically by Birkhoff’s “shooting method” [3] (1915) as well as numerically by Hill [7] in 1877, by Hénon [5, 6], and by Michalodimitrakis [9].

**Planar linear symmetries.** The planar Hamiltonian (1) is invariant under the double-symmetry given by the two commuting linear anti-symplectic involutions

3 $$\begin{aligned}{} & {} \rho _1 :T^* {\mathbb {R}}^2 \rightarrow T^*{\mathbb {R}}^2,\quad (q,p) \mapsto (q_1,-q_2,-p_1,p_2), \nonumber \\{} & {} \rho _2 :T^* {\mathbb {R}}^2 \rightarrow T^*{\mathbb {R}}^2,\quad (q,p) \mapsto (-q_1,q_2,p_1,-p_2). \end{aligned}$$

Their product $\rho _1 \circ \rho _2 = \rho _2 \circ \rho _1 = -\text {id}$ is symplectic. Geometrically, in the configuration space, the Hamiltonian $H_p$ is invariant under the reflections about the $q_1$- and $q_2$-axes, i.e., it is not possible to say whether we are going to the sun or away from it. The symplectic involutions ±id correspond to a rotation by 0 and $\pi$, respectively. Algebraically, $\rho _1, \rho _2$, and ±id form a Klein four-group, i.e.,

$$\begin{aligned} \Sigma _2 := \langle \rho _1, \rho _2 \mid \rho _1^2 = \rho _2^2 = (\rho _1 \circ \rho _2)^2 = \text {id} \rangle \cong {{\mathbb {Z}}}_2 \times {{\mathbb {Z}}}_2. \end{aligned}$$

For the planar problem, these symmetries are already all linear symmetries.

### Theorem A

$$ \{ \rho :T^* {\mathbb {R}}^2 \rightarrow T^* {\mathbb {R}}^2 \text { linear} \mid H_p \circ \rho = H_p,\ \rho ^2 = \textrm{id}, \text {and } \rho ^* \omega = \pm \omega \} = \Sigma _2. $$

**Spatial linear symmetries.** The spatial Hamiltonian (2) is invariant under the symplectic involution

$$\begin{aligned} \sigma :T^* {\mathbb {R}}^3 \rightarrow T^* {\mathbb {R}}^3,\quad (q_1,q_2,q_3,p_1,p_2,p_3) \mapsto (q_1,q_2,-q_3,p_1,p_2-p_3), \end{aligned}$$

which arises from the reflection at the ecliptic $\{q_3=0\}$. Moreover, the planar problem can be viewed as the restriction of this system to the fixed point set

$$\begin{aligned} \text {Fix}(\sigma ) = \{ (q_1,q_2,0,p_1,p_2,0) \}. \end{aligned}$$

Other linear symplectic symmetries are $-\sigma$ and $\pm \text {id}$, where $-\sigma$ corresponds to a rotation around the $q_3$-axis by $\pi$. Its fixed point set is $\text {Fix}(-\sigma ) = \{ ( 0,0,q_3,0,0,p_3 ) \}$, thus the $q_3$-axis is invariant under $-\sigma$. Four anti-symplectic symmetries are listed in Table 1.

**Table 1:** The linear anti-symplectic symmetries

Notation	Underlying geometry
$\rho _1(q,p)=(q_1,-q_2,q_3,-p_1,p_2,-p_3)$	Reflection at the $q_1q_3$-plane
$\rho _2(q,p)=(-q_1,q_2,q_3,p_1,-p_2,-p_3)$	Reflection at the $q_2q_3$-plane
$\overline{\rho _1}(q,p)=(q_1,-q_2,-q_3,-p_1,p_2,p_3)$	Rotation around the $q_1$-axis by $\pi$
$\overline{\rho _2}(q,p)=(-q_1,q_2,-q_3,p_1,-p_2,p_3)$	Rotation around the $q_2$-axis by $\pi$

Notice that all the maps $\sigma$, $\rho _1$, $\rho _2$, $\overline{\rho _1}$, and $\overline{\rho _2}$ leave $\text {Fix}(\sigma )$ invariant. Therefore, the two maps

$$\begin{aligned} \rho _1 |_{\text {Fix}(\sigma )} (q,p) = \overline{\rho _1} |_{\text {Fix}(\sigma )} (q,p)\quad \text {and}\quad \rho _2 |_{\text {Fix}(\sigma )} (q,p) = \overline{\rho _2} |_{\text {Fix}(\sigma )} (q,p) \end{aligned}$$

are exactly $\rho _1$ and $\rho _2$ from the planar problem. Furthermore, these eight linear symmetries form a group, which we denote by $\Sigma _3$. The group structure is given by Table 2.

**Table 2:** Group structure of $\Sigma _3$

$\circ$	id	−id	$-\sigma$	$\sigma$	$\rho _1$	$\rho _2$	$\overline{\rho _1}$	$\overline{\rho _2}$
id	id	−id	$-\sigma$	$\sigma$	$\rho _1$	$\rho _2$	$\overline{\rho _1}$	$\overline{\rho _2}$
−id	−id	id	$\sigma$	$-\sigma$	$\overline{\rho _2}$	$\overline{\rho _1}$	$\rho _2$	$\rho _1$
$-\sigma$	$-\sigma$	$\sigma$	id	−id	$\rho _2$	$\rho _1$	$\overline{\rho _2}$	$\overline{\rho _1}$
$\sigma$	$\sigma$	$-\sigma$	−id	id	$\overline{\rho _1}$	$\overline{\rho _2}$	$\rho _1$	$\rho _2$
$\rho _1$	$\rho _1$	$\overline{\rho _2}$	$\rho _2$	$\overline{\rho _1}$	id	$-\sigma$	$\sigma$	−id
$\rho _2$	$\rho _2$	$\overline{\rho _1}$	$\rho _1$	$\overline{\rho _2}$	$-\sigma$	id	−id	$\sigma$
$\overline{\rho _1}$	$\overline{\rho _1}$	$\rho _2$	$\overline{\rho _2}$	$\rho _1$	$\sigma$	−id	id	$-\sigma$
$\overline{\rho _2}$	$\overline{\rho _2}$	$\rho _1$	$\overline{\rho _1}$	$\rho _2$	−id	$\sigma$	$-\sigma$	id

This group is generated by $\{ \rho _1,\rho _2,\sigma \}$, and

$$\begin{aligned} \Sigma _3 \cong {{\mathbb {Z}}}_2 \times {{\mathbb {Z}}}_2 \times {{\mathbb {Z}}}_2 . \end{aligned}$$

By considering the four symplectic involutions $\pm \text {id}$, $\pm \sigma$, we see that, like in the planar case, a Klein four-group arises, namely as a sub-group of $\Sigma _3$. It is generated by $\{\pm \sigma \}$, and we denote it by $\Sigma _3^{\omega } \cong {{\mathbb {Z}}}_2 \times {{\mathbb {Z}}}_2$. Hence

$$\begin{aligned} \Sigma _2 \cong \Sigma _3^{\omega } \subset \Sigma _3. \end{aligned}$$

In the spatial problem, $\Sigma _3$ provides already all linear symmetries.

### Theorem B

$$ \{ \rho :T^* {\mathbb {R}}^3 \rightarrow T^* {\mathbb {R}}^3 \text { linear} \mid H \circ \rho = H,\ \rho ^2 = \textrm{id }, \text {and } \rho ^* \omega = \pm \omega \} = \Sigma _3. $$

The results of this paper are motivated by our work [1], in which we related Hill’s variational orbit from 1878 [8] to the Babylonian lunar periods by using Floquet multipliers and Conley–Zehnder indices. Therein the linear symmetries of Hill’s system and the invariance of Hill’s orbit were significant.

## Proof of Theorems A and B

First we show that *Theorem* B*implies Theorem* A, meaning that the spatial linear symmetries determine already the planar linear symmetries. This phenomenon is a remarkable property of Hill’s lunar system.

### Proof of Theorem A

Consider the projection map given by the restriction to $\text {Fix}(\sigma )$,

$$\begin{aligned} \pi :\Sigma _3 \rightarrow \Sigma _2,\quad \rho \mapsto \rho |_{\text {Fix}(\sigma )}. \end{aligned}$$

If $\rho \in \Sigma _3$ is symplectic or anti-symplectic, then $\rho |_{\text {Fix}(\sigma )}$ is a linear symplectic or anti-symplectic involution on $\text {Fix}(\sigma )$ as well, respectively. Moreover, $\rho |_{\text {Fix}(\sigma )}$ leaves the planar Hamiltonian $H_p$ (1) invariant. Therefore the map $\pi$ is well-defined.

While the map $\pi$ is not injective (since $\pi (\rho _1) = \pi (\overline{\rho _1})$), it is surjective. To see this, let $\rho \in \Sigma _2$. If $\rho$ is symplectic, then a symplectic extension is given by $q_3 \mapsto q_3$ and $p_3 \mapsto p_3$. If $\rho$ is anti-symplectic, then an anti-symplectic extension is given by $q_3 \mapsto -q_3$ and $p_3 \mapsto p_3$. Theorem A thus follows from Theorem B.

$$\square $$

In order to prove Theorem B, we first recall some basic prerequisites from linear symplectic geometry. Consider the standard symplectic vector space $({\mathbb {R}}^{2n},\omega _0)$ with

$$\begin{aligned} \omega _0 (v,w) : = \langle Jv,w \rangle = v^T J^T w = \langle v,J^Tw \rangle \quad \text { for all }v,w \in {\mathbb {R}}^{2n}, \end{aligned}$$

where

$$\begin{aligned} J = \begin{pmatrix} 0 &{}\quad I_n\\ -I_n &{}\quad 0 \end{pmatrix} \end{aligned}$$

with respect to the splitting ${\mathbb {R}}^{2n} = {\mathbb {R}}^n \times {\mathbb {R}}^n$. Note that $J^2 = -I_{2n}$ and $J^T=J^{-1}=-J$. A linear isomorphism $\Psi$ of $({\mathbb {R}}^{2n},\omega _0)$ is called symplectic if $\omega _0 (\Psi v, \Psi w) = \omega _0 (v,w) \text { for all }v,w \in {\mathbb {R}}^{2n},$ which is equivalent to $\Psi ^T J \Psi = J$. The set of symplectic matrices in ${\mathbb {R}}^{2n}$ is denoted by

$$\begin{aligned} \text {Sp}(n) = \{ \Psi :({\mathbb {R}}^{2n},\omega _0) \rightarrow ({\mathbb {R}}^{2n},\omega _0) \text { linear isomorphism } | \text { } \Psi ^T J \Psi = J \}. \end{aligned}$$

It is easy to show that if $\Psi ,\Phi \in \text {Sp}(n)$, then $\Psi \Phi , \Psi ^{-1}$, $\Psi ^T \in \text {Sp}(n)$ and also $J \in \text {Sp}(n)$. In particular, $\text {Sp}(n)$ is a group under matrix multiplication. Moreover, a $2n \times 2n$ matrix, which is written as

4 $$\begin{aligned} \begin{pmatrix} A &{}\quad B\\ C &{}\quad D \end{pmatrix} \end{aligned}$$

with respect to the splitting ${\mathbb {R}}^{2n} = {\mathbb {R}}^n \times {\mathbb {R}}^n$, is symplectic if and only if

5 $$\begin{aligned} A^T C , B^T D \text { are symmetric and } A^T D - C^T B = I_n. \end{aligned}$$

Its inverse is given by

$$\begin{aligned} \begin{pmatrix} D^T &{}\quad -B^T\\ -C^T &{}\quad A^T \end{pmatrix}. \end{aligned}$$

We denote the set of anti-symplectic matrices in ${\mathbb {R}}^{2n}$ by

$$\begin{aligned} \text {Sp}^-(n) = \{ \Psi :({\mathbb {R}}^{2n},\omega _0) \rightarrow ({\mathbb {R}}^{2n},\omega _0) \text { linear isomorphism } | \text { } \Psi ^T J \Psi = - J \}, \end{aligned}$$

which is not a group since for $\Psi ,\Phi \in \text {Sp}^-(n)$, the multiplication $\Psi \Phi$ is symplectic. Nevertheless $\Psi ^{-1},\Psi ^T \in \text {Sp}^-(n)$ and $-J \in \text {Sp}^-(n)$. A $2n \times 2n$ matrix given in the block form (4) is anti-symplectic if and only if

6 $$\begin{aligned} A^T C , B^T D \text { are symmetric and } A^T D - C^T B = -I_n. \end{aligned}$$

The inverse matrix is given by

$$\begin{aligned} \begin{pmatrix} -D^T &{}\quad B^T\\ C^T &{}\quad -A^T \end{pmatrix}. \end{aligned}$$

### Proof of Theorem B

Let $\rho$ be a linear symmetry. We prove the theorem in three steps where the first one is obvious.

**Step 1.** The Hamiltonian (2) is the sum of

$$\begin{aligned} H_2(q,p) = \frac{1}{2}|p|^2 + p_1q_2 - p_2q_1 - q_1^2 + \frac{1}{2}q_2^2 + \frac{1}{2}q_3^2 \quad \text {and} \quad H_{-1}(q,p) = - \frac{1}{|q|}, \end{aligned}$$

where $H_2$ is homogeneous of degree 2 and $H_{-1}$ is homogeneous of degree $-1$. Hence $H_2 \circ \rho = H_2$ and $H_{-1} \circ \rho = H_{-1}.$

**Step 2.** *The matrix form of* $\rho$ *with respect to the splitting* ${\mathbb {R}}^6 = {\mathbb {R}}^3 \times {\mathbb {R}}^3$ *and to the coordinates* $(q_1,q_2,q_3,p_1,p_2,p_3)$ *is*

$$\begin{aligned} {\left\{ \begin{array}{ll} \begin{pmatrix} A &{}\quad 0\\ C &{}\quad A \end{pmatrix},\quad &{}A \in O(3),\quad A=A^T,\quad C=-C^T,\quad AC=-CA \\ {} &{}\qquad \text { if }\rho \text { is symplectic,}\\ \\ \begin{pmatrix} A &{}\quad 0\\ C &{}\quad -A \end{pmatrix},\quad &{}A \in O(3),\quad A=A^T,\quad C=C^T,\quad AC=CA \\ {} &{}\qquad \text { if }\rho \text { is anti-symplectic.} \end{array}\right. } \end{aligned}$$

To see that, we write $\rho$ in matrix form

$$\begin{aligned} \begin{pmatrix} A &{}\quad B\\ C &{}\quad D \end{pmatrix}, \end{aligned}$$

with respect to the coordinates $(q_1,q_2,q_3,p_1,p_2,p_3)$, where $A,B,C,D \in \text {Mat}(3,{\mathbb {R}}).$ The $\rho$-invariance of $H_{-1}$ yields

$$\begin{aligned} |Aq + Bp| = |q|\quad \forall q,p. \end{aligned}$$

For fixed *p*, we take *q* with |*q*| very small and find $Bp=0$. Hence

$$\begin{aligned} B = 0,\quad A \in O(3). \end{aligned}$$

Next, the $\rho$-invariance of $H_2$ yields for $q=0$,

$$\begin{aligned} |Dp| = |p|\quad \forall p, \end{aligned}$$

whence also $D \in O(3).$ Since $\rho$ is an involution, we obtain

$$\begin{aligned} \rho \circ \rho = \begin{pmatrix} A^2 &{} \quad 0\\ CA + DC &{}\quad D^2 \end{pmatrix} = \begin{pmatrix} I_3 &{}\quad 0\\ 0 &{}\quad I_3 \end{pmatrix}, \end{aligned}$$

and with $AA^T = DD^T = I_3$, we obtain

7 $$\begin{aligned} A = A^T,\quad D = D^T,\quad CA + DC = 0. \end{aligned}$$

If $\rho$ is symplectic, then (7) and the linear symplectic relations (5) imply

$$\begin{aligned} AC=A^TC=C^TA,\quad A^TD=AD=I_3. \end{aligned}$$

With $A^2=I_3$, we have

$$\begin{aligned} D=A, \end{aligned}$$

and therefore

$$\begin{aligned} 0 = CA + DC = CA + AC = CA + C^TA = (C+C^T)A. \end{aligned}$$

Since det$(A)=\pm 1$, the matrix *C* is skew-symmetric and this proves the first assertion of the second step.

If $\rho$ is anti-symplectic, then by (7) and the linear anti-symplectic conditions (6), we obtain

$$\begin{aligned} AC=A^TC=C^TA,\quad A^T D = AD = -I_3, \end{aligned}$$

hence

$$\begin{aligned} D=-A, \end{aligned}$$

and

$$\begin{aligned} 0 = CA + DC = CA - AC = CA - C^TA = (C-C^T)A. \end{aligned}$$

Therefore the matrix *C* is symmetric and the second assertion follows.

**Step 3.** *In both cases, the matrix* *C* *is the zero matrix and*

$$\begin{aligned} \begin{aligned} {\left\{ \begin{array}{ll} \rho \in \{ \pm \sigma , \pm \text{ id } \} &{}{} \text{ if } \rho \text{ is } \text{ symplectic, }\\ \rho \in \{\rho _1, \rho _2\, \overline{\rho _1}, \overline{\rho _2}\} &{}{} \text{ if } \rho \text{ is } \text{ anti-symplectic. } \end{array}\right. } \end{aligned} \end{aligned}$$

In both cases, *A* is of the form

$$\begin{aligned} A = \begin{pmatrix} a &{}\quad d &{}\quad e\\ d &{}\quad b &{}\quad f\\ e &{}\quad f &{}\quad c \end{pmatrix}. \end{aligned}$$

Since $A^2 = I_3$ and $A \in O(3)$, we have

8 $$\begin{aligned} A^2 = \begin{pmatrix} a^2 + d^2 + e^2 &{}\quad ad+bd+ef &{}\quad ae+df+ce\\ ad+bd+ef &{}\quad d^2 + b^2 + f^2 &{}\quad de+bf+cf\\ ae+df+ce &{}\quad de+bf+cf &{}\quad e^2 + f^2 + c^2 \end{pmatrix} = \begin{pmatrix} 1 &{}\quad 0 &{}\quad 0\\ 0 &{}\quad 1 &{}\quad 0\\ 0 &{}\quad 0 &{}\quad 1 \end{pmatrix} \end{aligned}$$

and

$$\begin{aligned} \det (A)= abc + 2def - af^2 - be^2 - cd^2 = \pm 1. \end{aligned}$$

If $\rho$ is symplectic, then $\rho (q,p)$ is of the form

$$\begin{aligned} \begin{pmatrix} a &{}\quad d &{}\quad e &{}\quad 0 &{}\quad 0 &{}\quad 0\\ d &{}\quad b &{}\quad f &{}\quad 0 &{}\quad 0 &{}\quad 0\\ e &{}\quad f &{}\quad c &{}\quad 0 &{}\quad 0 &{}\quad 0\\ 0 &{}\quad c_1 &{}\quad c_2 &{}\quad a &{}\quad d &{}\quad e\\ -c_1 &{}\quad 0 &{}\quad c_3 &{}\quad d &{} \quad b &{}\quad f\\ -c_2 &{}\quad -c_3 &{}\quad 0 &{}\quad e &{}\quad f &{}\quad c \end{pmatrix} \cdot \begin{pmatrix} q_1\\ q_2\\ q_3\\ p_1\\ p_2\\ p_3 \end{pmatrix} = \begin{pmatrix} aq_1 + dq_2 + eq_3\\ dq_1 + bq_2 + fq_3\\ eq_1 + fq_2 + cq_3\\ c_1q_2 + c_2q_3 + ap_1 + dp_2 + ep_3\\ -c_1q_1 + c_3q_3 + dp_1 + bp_2 + fp_3\\ -c_2q_1 - c_3q_2 + ep_1 + fp_2 + cp_3 \end{pmatrix}. \end{aligned}$$

The equation $AC=-CA$ yields

9 $$\begin{aligned}{} & {} \begin{pmatrix} -dc_1 - ec_2 &{}\quad ac_1 - ec_3 &{}\quad ac_2 + dc_3\\ -bc_1 - fc_2 &{}\quad dc_1 - fc_3 &{}\quad dc_2 + bc_3\\ -fc_1 - cc_2 &{}\quad ec_1 - cc_3 &{}\quad ec_2 + fc_3 \end{pmatrix}\nonumber \\ {}{} & {} = \begin{pmatrix} -dc_1 - ec_2 &{}\quad -bc_1 - fc_2 &{}\quad -fc_1 - cc_2\\ ac_1 - ec_2 &{} \quad dc_1 - fc_3 &{}\quad ec_1 - cc_3\\ ac_2 + dc_3 &{}\quad dc_2 + bc_3 &{}\quad ec_2 + fc_3 \end{pmatrix}, \end{aligned}$$

and therefore

10 $$\begin{aligned} ac_1 - ec_3 = -bc_1 - fc_2,\quad ac_2 + dc_3 = -fc_1 - cc_2,\quad dc_2 + bc_3 = ec_1 - cc_3.\nonumber \\ \end{aligned}$$

In view of the $\rho$-invariance of $H_2$, we compare

$$\begin{aligned} H_2(q,p) = \frac{1}{2}|p|^2 + p_1q_2 - p_2q_1 - q_1^2 + \frac{1}{2}q_2^2 + \frac{1}{2}q_3^2 \end{aligned}$$

with $H_2\big ( \rho (q,p) \big )$, which is

$$\begin{aligned}&\frac{1}{2}|p|^2 + p_1q_2\left( ac_1 - ec_3 + ab - d^2\right) - p_2q_1\left( bc_1 + fc_2 + ab - d^2\right) \\&\quad - q_1^2 \left( \frac{1}{2}\left( -c_1^2 -c_2^2 - d^2 - e^2 \right) - ac_1 + a^2 \right) \\&\quad + \frac{1}{2}q_2^2 \left( c_1^2 + c_3^2 + 2bc_1 - 2d^2 + b^2 + f^2\right) \\&\quad + \frac{1}{2}q_3^2 \left( c_2^2 + c_3^2 + 2fc_2 - 2ec_3 - 2e^2 + f^2 + c^2\right) \\&\quad + p_1q_1 \left( -dc_1 - ec_2 \right) + p_2q_2 \left( dc_1 - fc_3 \right) + p_3q_3 \left( ec_2 + fc_3 \right) \\&\quad + p_1q_3 \left( ac_2 + dc_3 + af - de\right) + p_3q_1 \left( -fc_1 - cc_2 + de - af \right) \\&\quad + p_2q_3 \left( dc_2 + bc_3 + df - be \right) + p_3q_2 \left( ec_1 - cc_3 + be - df \right) \\&\quad + q_1q_2 \left( c_2c_3 + 2dc_1 - 2ad + bd + ef \right) \\&\quad + q_1q_3 \left( -c_1c_3 + dc_2 + ec_1 - ac_3 - 2ae + df + ce \right) \\&\quad + q_2q_3 \left( c_1c_2 + fc_1 + bc_2 - dc_3 - 2de + bf + cf \right) . \end{aligned}$$

By the coefficients of $p_i q_i$ for $i=1,2,3$, we immediately have that the diagonal entries of *AC* in (9) are all zero. To see that the other entries of *AC* are also all zero, we set equal the coefficients of $p_1q_2$ with $p_2q_1$, of $p_1q_3$ with $p_3q_1$, of $p_2q_3$ with $p_3q_2$, and use (10), which imply

$$\begin{aligned} ac_1 - ec_3{} & {} = -bc_1 - fc_2=0,\quad ac_2 + dc_3 = -fc_1 - cc_2=0,\\ dc_2 + bc_3{} & {} = ec_1 - cc_3=0. \end{aligned}$$

Hence $AC=0$ and thus $C=0$. By the coefficients of $p_1q_2$ and $p_2q_1$,

$$\begin{aligned} ab-d^2 = 1, \end{aligned}$$

which means that $a \ne 0$ and $b \ne 0$. In view of $A^2 = I_3$ in (8), the two equations $ad+bd+ef=0$ and $ae+df+ce=0$ together with the coefficients of $q_1q_2$ and $q_1q_3$ imply $ad=ae=0$. Since $a \ne 0$, we obtain

$$\begin{aligned} d=e=0 . \end{aligned}$$

Furthermore, by the coefficient of $p_1q_3$, we have $af=de=0$, hence $f=0$. Together with the coefficients from the second until the fourth lines, we obtain

$$\begin{aligned} ab=1,\quad a^2=b^2=c^2=1 , \end{aligned}$$

which correspond to $\pm \sigma$ and $\pm ~id$.

If $\rho$ is anti-symplectic, then $\rho (q,p)$ is of the form

$$\begin{aligned}{} & {} \begin{pmatrix} a &{}\quad d &{}\quad e &{}\quad 0 &{}\quad 0 &{}\quad 0\\ d &{}\quad b &{}\quad f &{}\quad 0 &{}\quad 0 &{}\quad 0\\ e &{}\quad f &{}\quad c &{}\quad 0 &{}\quad 0 &{}\quad 0\\ c_1 &{}\quad c_2 &{}\quad c_3 &{}\quad -a &{}\quad -d &{}\quad -e\\ c_2 &{}\quad c_4 &{}\quad c_5 &{}\quad -d &{}\quad -b &{}\quad -f\\ c_3 &{}\quad c_5 &{}\quad c_6 &{}\quad -e &{}\quad -f &{}\quad -c \end{pmatrix} \cdot \begin{pmatrix} q_1\\ q_2\\ q_3\\ p_1\\ p_2\\ p_3 \end{pmatrix} \\ {}{} & {} = \begin{pmatrix} aq_1 + dq_2 + eq_3\\ dq_1 + bq_2 + fq_3\\ eq_1 + fq_2 + cq_3\\ c_1q_1 + c_2q_2 + c_3q_3 - ap_1 - dp_2 - ep_3\\ c_2q_1 + c_4q_2 + c_5q_3 - dp_1 - bp_2 - fp_3\\ c_3q_1 + c_5q_2 + c_6q_3 - ep_1 - fp_2 - cp_3 \end{pmatrix}. \end{aligned}$$

The equation $AC=CA$ yields that

$$\begin{aligned} \begin{pmatrix} ac_1 + dc_2 + ec_3 &{}\quad ac_2 + dc_4 + ec_5 &{}\quad ac_3 + dc_5 + ec_6\\ dc_1 + bc_2 + fc_3 &{}\quad dc_2 + bc_4 + fc_5 &{}\quad dc_3 + bc_5 + fc_6\\ ec_1 + fc_2 + cc_3 &{}\quad ec_2 + fc_4 + cc_5 &{}\quad ec_3 + fc_5 + cc_6 \end{pmatrix} \end{aligned}$$

equals

$$\begin{aligned} \begin{pmatrix} ac_1 + dc_2 + ec_3 &{}\quad dc_1 + bc_2 + fc_3 &{}\quad ec_1 + fc_2 + cc_3\\ ac_2 + dc_4 + ec_5 &{}\quad dc_2 + bc_4 + fc_5 &{}\quad ec_2 + fc_4 + cc_5\\ ac_3 + dc_5 + ec_6 &{}\quad dc_3 + bc_5 + fc_6 &{}\quad ec_3 + fc_5 + cc_6 \end{pmatrix}. \end{aligned}$$

Therefore

$$\begin{aligned} ac_2 + dc_4 + ec_5&= dc_1 + bc_2 + fc_3,\\ ac_3 + dc_5 + ec_6&= ec_1 + fc_2 + cc_3,\\ dc_3 + bc_5 + fc_6&= ec_2 + fc_4 + cc_5. \end{aligned}$$

Now $$H_2\big ( \rho (q,p) \big )$$ is

$$\begin{aligned}&\frac{1}{2}|p|^2 + p_1q_2\left( -ac_2 - dc_4 - ec_5 + d^2 - ab\right) - p_2q_1\left( dc_1 + bc_2 + fc_3 + d^2 - ab\right) \\&\quad - q_1^2 \left( \frac{1}{2}\left( -c_1^2 -c_2^2 - d^2 - e^2 \right) - dc_1 + ac_2 + a^2 \right) \\&\quad + \frac{1}{2}q_2^2 \left( c_2^2 + c_4^2 + c_5^2 + 2bc_2 - 2dc_4 - 2d^2 + b^2 + f^2\right) \\&\quad + \frac{1}{2}q_3^2 \left( c_3^2 + c_5^2 + c_6^2 + 2fc_3 - 2ec_5 - 2e^2 + f^2 + c^2\right) \\&\quad + p_1q_1 \left( -ac_1 - dc_2 - ec_3 \right) + p_2q_2 \left( -dc_2 - bc_4 - fc_5 \right) \\ {}&\quad + p_3q_3 \left( -ec_3 - fc_5 - cc_6 \right) \\&\quad + p_1q_3 \left( -ac_3 - dc_5 - ec_6 + de - af\right) + p_3q_1 \left( -ec_1 - fc_2 - cc_3 + af - de \right) \\&\quad + p_2q_3 \left( -dc_3 - bc_5 - fc_6 + be - df \right) + p_3q_2 \left( -ec_2 - fc_4 - cc_5 + df - be \right) \\&\quad + q_1q_2 \left( c_1c_2 + c_2c_4 + c_3c_5 + bc_1 - ac_4 - 2ad + bd + ef \right) \\&\quad + q_1q_3 \left( c_1c_3 + c_2c_5 + c_3c_6 + fc_1 + dc_3 - ec_2 - ac_5 - 2ae + df + ce \right) \\&\quad + q_2q_3 \left( c_2c_3 + c_4c_5 + c_5c_6 + fc_2 + bc_3 - ec_4 - dc_5 - 2de + bf + cf \right) . \end{aligned}$$

In a similar way to the symplectic case, we find that $C=0$. In view of $A^2 = I_3$ in (8), the three equations $ad+bd+ef=0$, $ae+df+ce=0$, and $de + bf + cf=0$ together with the coefficients of $q_1q_2$, $q_1q_3$, $q_2q_3$ imply $ad=ae=de=0$. Since the coefficients of $p_1q_3$ and $p_3q_1$ yield $de=af$, we have

$$\begin{aligned} ad=ae=af=0. \end{aligned}$$

Suppose that $a = 0$, then in view of the coefficients of $q_1^2$, we see that $d^2 + e^2 = -2$ which is a contradiction. Hence

$$\begin{aligned} a \ne 0,\quad d=e=f=0. \end{aligned}$$

By the first four lines, we obtain

$$\begin{aligned} ab = -1,\quad a^2 = b^2 = c^2 = 1, \end{aligned}$$

which correspond to $\rho _1,\rho _2,\overline{\rho _1}$, and $\overline{\rho _2}$. $\square$
